# Melanoma prevalence: can medical literacy overcome the rise in UV radiation? United States as a case study

**DOI:** 10.3389/fpubh.2025.1636571

**Published:** 2025-08-06

**Authors:** Yuval Arbel, Yifat Arbel, Netanel Kerner, Miryam Kerner

**Affiliations:** ^1^Sir Harry Solomon School of Economics and Management, Western Galilee College, Acre, Israel; ^2^Department of Mathematics, Bar Ilan University, Ramat Gan, Israel; ^3^Faculty of Medicine, Hebrew University of Jerusalem, Jerusalem, Israel; ^4^The Ruth and Bruce Rapoport Faculty of Medicine, Technion – Israel Institute of Technology, Haifa, Israel; ^5^Department of Dermatology, Emek Medical Center, Afula, Israel

**Keywords:** melanoma prevalence, health literacy, knowledge spillover, UV radiation, population size

## Abstract

**Background:**

Recent literature shows melanoma prevalence is steadily increasing, mainly due to UV radiation exposure, especially in Caucasians. Skin cancer causes $8.9 billion in direct annual cost and unmeasured indirect costs, but can be prevented by avoiding sun exposure, using protective creams, and regular dermatologists visits for suspicious nevi.

**Methods:**

Using CDC data on non-Hispanic Caucasians, we conducted an Analysis of Variance (ANOVA) to examine the differences in age-adjusted melanoma incidence rates per 100,000. Quadratic Poisson, Ordinary Least Square (OLS) and Two-Stage Least Squares (TSLS) regressions were employed, with UV index and education levels—measured by the percentage of the population holding at least a bachelor’s degree—as key explanatory variables.

**Conclusion:**

From a public policy standpoint, raising awareness about sun exposure protection and encouraging regular examinations by professional dermatologists, especially in sparsely populated areas with high UV radiation—could be crucial in reducing the remarkable costs associated with melanoma morbidity and treatment.

## Highlights

- Projected new melanoma cases have doubled over a 23-year period.
- Projected new melanoma cases drop from 800–835 to 340–380 as population rises, despite higher UV radiation levels in larger states like California.
- The protective effect of education against melanoma is supported empirically:
- A 12–16% increase in education level offsets the 23-year melanoma growth rate.
- A 25–30% increase in education level offsets a 1% rise in UV exposure.

## 1 Introduction

Melanoma is a skin cancer, which usually develops on sun-exposed skin, such as the arms, back, face, and legs, but can also occur in the eyes or rarely in internal areas like the nose or throat. Most cases are linked to ultraviolet (UV) light from sunlight or tanning beds. In the U.S., sunburn prevalence is high, with 37.1% of adults and 55.8% of youth reporting at least one sunburn in the past year (1). Limiting UV exposure can help reduce the risk of melanoma (1–3).

Skin cancer also incurs remarkable direct and indirect costs of $8.9 billion annually (in addition to unmeasurable indirect costs). According to the CDC report on skin cancer in the United States, nearly 6 million people are treated for skin cancer each year (4, 5).

This study investigates the social and environmental factors contributing to melanoma incidence in the United States. Our research question is whether education can mitigate UV-related melanoma risk. The analysis explores how melanoma rates are influenced by medical literacy—proxied by population size and education levels—along with UV radiation and temporal trends. Using CDC data on non-Hispanic Caucasian males and females, the study applies quadratic Poisson regression and Two-Stage Least Squares (TSLS) over a 23-year period. The initial model uses the count of new melanoma cases as the dependent variable, with population size, state fixed effects (as proxies for UV exposure), and time as independent variables. To address overdispersion, extended models substitute the dependent variable with age-adjusted annual melanoma cases per 100,000 people and replace state dummies with direct UV index values and the share of the educated population. Building on prior research linking population size to knowledge spillovers and stronger health systems, the study finds that higher education levels—representing human capital—can mitigate environmental health risks like UV-related melanoma.

Results corroborate a strong connection between state dummies and UV radiation levels, showing that after accounting for these levels, projected new melanoma cases have doubled over the past 23 years. However, higher population and education levels are linked to fewer cases, even in high-UV states. Robustness tests reveal that the Poisson model may not be adequate due to overdispersion. Extended models confirm that a 12–16% rise in the educated population offsets long-term melanoma growth, and a 25–30% increase counters a 1% rise in UV exposure. These findings highlight the role of education in reducing melanoma risk.

## 2 Description of data

The data for this study were generated, among other sources, from the CDC website and focus exclusively on non-Hispanic White males and females in the United States, as this demographic is the most susceptible to melanoma skin cancer. In 2022, the total population of non-Hispanic Whites in the United States was 197,639,521.

More specifically, the data were drawn from the following sources:

1. CDC: United States Cancer Statistics: Data Visualizations: Trends. Available at: https://gis.cdc.gov/Cancer/USCS/#/Trends/ (6)
2. World Population Review UV Index by State. Available at: https://worldpopulationreview.com/state-rankings/uv-index-by-state (7).
3. US Bureau of Census. Data available at: https://www.census.gov/data.html (8).

The datasets and script for replications of the outcomes obtained are given in the following link: https://github.com/YuvalArbel1234/Frontiers_Melanoma_Human_Capital/releases

The combined dataset consists of 2,325 observations, each representing statewide-level data for a specific year, spanning from 1999 to 2022 (a total of 23 years). It is clear that the sample includes data from all 51 U.S. states. In 47 states, the sample contains 46 observations per state (one for Caucasian females and one for Caucasian males over 23 years). Two states have 42 observations each (spanning 21 years), one state (the District of Columbia) has 41 observations (covering 20–21 years), and one state (Mississippi) has 38 observations (covering 19 years). The total is calculated as 47×46+2×42+41+38=2,325 .

Table 1A reports the descriptive statistics of variables that are latter incorporated in the empirical model. Table 1B gives the covariance matrix between the variables. The average number of new melanoma cases per state is 631 per annum and the standard deviation is 702 (CaseCount). The 99% confidence interval is [594, 669]. The average population per state is 1,969,797 persons and the standard deviation is 1,737,117 persons (Population).

**Table 1:** Description of data.

A. Descriptive statistics (*N* = 2,325)					
Variable	Description	Mean	Std. Dev.	Min	Max
Case Count	Number of annual new melanoma cases in the state.	631	702	16	5,397
Population	Population of non-Hispanic Caucasians in the state.	1,969,797	1,735,117	79,874	8,288,635
(Year−1999)	0,1,2,⋯,22 for Year=1999,2000,2001,⋯,2021	11	7	0	22

B. Covariance matrix (*N* = 2,325)				
	Case Count	$\text{Populatio}n^{2}$	Population	(Year−1999)
Case Count	1.0000			
				
$\text{Populatio}n^{2}$	0.8548***	1.0000		
	(<0.01)			
Population	0.8613***	0.9439***	1.0000	
	(<0.01)	(<0.01)		
(Year−1999)	0.1847***	−0.0056	0.0000	1.0000
	(<0.01)	(0.7879)	(0.9991)	

The population consists solely of non-Hispanic Caucasians, who are the group most vulnerable to melanoma skin cancer. In 2022, the population of non-Hispanic Caucasians in the United States totaled 197,639,521 individuals. The numbers in parentheses represent the *p*-values for testing the null hypothesis of no Pearson correlation. ***p < 0.01.

Referring to the correlation matrix, there is a strong positive Pearson correlation of 0.86 between the Case Count and Population variables. This indicates that the Population variable explains 86% of the standard deviation in the Case Count variable. Since the null hypothesis of zero correlation between Case Count and (Year−1999) cannot be rejected, there is also a positive time trend in the number of new melanoma cases. Lastly, as expected, there is a high Pearson correlation of 0.94 between the Population and Population squared variables.

## 3 Methodology

Consider the following empirical model:

*Model A*: Basic Poisson Regression

(1) $$\begin{array}{c} \text{Case Count}=\alpha_{1}\text{Populatio}n^{2}+\alpha_{2}\text{Population}\\ +\alpha_{3}+\alpha_{4}(\text{Year}-1999)+A\beta^{T}+\mu_{A} \end{array}$$

In this specification, *Case Count* is the dependent variable. Case Count is the dependent variable, The independent variables include: Population_sq (population squared), Population , and (Year−1999) , The parameters $\alpha_{1},\alpha_{2},\alpha_{3},\alpha_{4}$ are are to be estimated. A denotes a matrix of U.S. state dummy variables (with Alabama serving as the base category), and $\beta^{T}$ is the corresponding column vector of coefficients, and $\mu_{1}$ is the random disturbance term.

Poisson regression is employed to model the counts of an event’s occurrences [e.g., (9), pp. 595–600]. Applications of the Poisson distribution include diverse events such as the pattern of hits by buzz bombs launched on London during World War II (10), the number of soldiers killed by horse kicks in the Prussian army (11), and telephone calls resulting in a wrong number (12). It has also been used to model disease incidence, most commonly over time but sometimes in spatial contexts.

According to Chiang and Wainwright (13), the general form of a quadratic function is $y=ax^{2}+\mathrm{bx}+c\;$ , where (a≠0) , and its second derivative is 2a . Since this second derivative always has the same sign as the coefficient a , the function forms a U-shaped curve with a global minimum at ( $\frac{-b}{2a}\;$ , $\frac{-b^{2}+4\mathrm{ac}}{4a}$ ) when a > 0, and an inverted U-shaped curve with a global maximum at the same point when a < 0.

In this study, y represents the number of annual new melanoma cases in the state. ( Case Count ) in Equation 1; $x^{2}$ corresponds to Population_sq , and x represents Population , with coefficients $a=\alpha_{1}$ ; $b=\alpha_{2}$ and $c=\alpha_{3}$ (Equation 1). Compared to the linear model, the quadratic model offers flexibility by not assuming a strictly monotonic relationship between population and the number of annual new melanoma cases.

One limitation of the quadratic model is the high collinearity between $\text{Populatio}n^{2}$ and *Population*, with a Pearson correlation coefficient of 0.9439 (see Table 1B). As noted by Arbel et al. (14), such high collinearity can distort estimation results and lead to misleading conclusions. This issue can be addressed by excluding either *Population* or *Population sq*, leading to the following alternative specifications:

*Model A1*: Basic Poisson Regression (Partial Quadratic Model)

(1a) $$\text{Case Count}=\alpha_{1}\text{Populatio}n^{2}+\alpha_{3}+\alpha_{4}(\text{Year}-1999)+A\beta^{T}+\mu_{A1}$$

*Model A2*: Basic Poisson Regression (Linear Model)

(1b) $$\text{Case Count}=\alpha_{2}\text{Population}+\alpha_{3}+\alpha_{4}(\text{Year}-1999)+A\beta^{T}+\mu_{A2}$$

Finally, note that the variable (Year−1999)=0,1,2,⋯,22 in Equations 1, 1a, and 1b captures the linear time trend. To relax the linearity assumption and permit non-linear relationships, an alternative version of the empirical models replaces (Year−1999) with dummy variables DUM_Year1999,DUM_Year2000;⋯;DUM_Year2023 where each dummy variable equals 1 if (Year−1999)=0,1,2,⋯,22 and zero otherwise.

## 4 Results

Table 2 reports the regression outcomes of the model defined by Equations 1a and 1b with and without linear time trend. Columns (1) and (2) give the structural coefficients and Columns (3) and (4)—the exponent of the structural coefficients. It may be readily verified that exp(·)−1 yields the percentage of growth [e.g., (15)]. For example, a coefficient of 0.0317 results in exp(0.0317)−1=3.2% representing the percentage of growth.

**Table 2:** Poisson regressions.

	(1)	(2)	(3)	(4)
Coefficient	Structural	Structural	Exp(Structural)	Exp(Structural)
Variables	Case Count	Case Count	Case Count	Case Count
$\text{Populatio}n^{2}$	−1.17 × 10^−14^***	−1.37 × 10^−14^***	1***	1***
	(4.85 × 10^−16^)	(4.97 × 10^−16^)	(4.85 × 10^−16^)	(4.97 × 10^−16^)
(Year−1999)	0.0317***	-	1.032***	-
	(0.0001281)	-	(0.0001323)	-
Constant	5.872***	5.770***	355.0***	320.4***
	(0.0069542)	(0.0084109)	(2.468997)	(2.69516)
States fixed effects				
Alaska	−2.576***	−2.581***	0.0761***	0.0757***
	(0.0245089)	(0.0245106)	(0.0018647)	(0.0018555)
Arizona	0.372***	0.374***	1.451***	1.454***
	(0.0086053)	(0.0086058)	(0.0124876)	(0.0125092)
Arkansas	−0.657***	−0.660***	0.518***	0.517***
	(0.0112552)	(0.0112563)	(0.0058351)	(0.0058193)
California	2.610***	2.722***	13.60***	15.22***
	(0.0286444)	(0.0293484)	(0.3896329)	(0.4465665)
Colorado	0.0820***	0.0833***	1.085***	1.087***
	(0.0091738)	(0.0091742)	(0.0099574)	(0.0099716)
Connecticut	−0.176***	−0.178***	0.838***	0.837***
	(0.0097552)	(0.0097558)	(0.0081775)	(0.008162)
Delaware	−1.378***	−1.383***	0.252***	0.251***
	(0.0145902)	(0.0145929)	(0.0036771)	(0.0036596)
District of Columbia	−3.086***	−3.091***	0.0457***	0.0455***
	(0.032643)	(0.0326449)	(0 0.0014911)	(0.001484)
Florida	1.998***	2.054***	7.374***	7.799***
	(0.0156659)	(0.0159839)	(0 0.115526)	(0.1246563)
Georgia	0.819***	0.829***	2.268***	2.290***
	(0.0083362)	(0.0083544)	(0 0.0189089)	(0.0191322)
Hawaii	−1.308***	−1.313***	0.270***	0.269***
	(0.0141932)	(0.0141961)	(0 0.0038374)	(0.0038187)
Idaho	−0.884***	−0.889***	0.413***	0.411***
	(0.0121459)	(0.0121483)	(0 0.0050157)	(0.0049955)
Illinois	0.998***	1.025***	2.712***	2.788***
	(0.0104466)	(0.0105663)	(0 0.0283292)	(0.0294559)
Indiana	0.299***	0.294***	1.349***	1.342***
	(0.0093159)	(0.0093284)	(0 0.0125646)	(0.0125152)
				
Iowa	−0.236***	−0.238***	0.790***	0.789***
	(0.0099181)	(0.0099185)	(0 0.0078332)	(0.0078216)
Kansas	−0.461***	−0.463***	0.631***	0.629***
	(0.0105815)	(0.0105826)	(0 0.0066752)	(0.0066582)
Kentucky	0.139***	0.141***	1.149***	1.151***
	(0.0090604)	(0.0090611)	(0 0.0104126)	(0.0104325)
Louisiana	−0.369***	−0.371***	0.691***	0.690***
	(0.0103044)	(0.0103047)	(0 0.0071235)	(0.0071138)
Maine	−1.009***	−1.013***	0.365***	0.363***
	(0.012694)	(0.0126964)	(0 0.0046284)	(0.0046092)
Maryland	0.275***	0.275***	1.317***	1.316***
	(0.0087544)	(0.0087544)	(0.0115256)	(0.0115247)
Massachusetts	0.407***	0.414***	1.502***	1.513***
	(0.0088068)	(0.0088177)	(0.0132252)	(0.0133432)
Michigan	0.854***	0.878***	2.348***	2.405***
	(0.0100132)	(0.0101051)	(0.0235131)	(0.0243024)
Minnesota	0.460***	0.465***	1.584***	1.591***
	(0.0085552)	(0.0085594)	(0.01355)	(0.01362)
Mississippi	−0.705***	−0.715***	0.494***	0.489***
	(0.0119588)	(0.0119632)	(0.005911)	(0.0058516)
Missouri	0.143***	0.149***	1.153***	1.161***
	(0.0092421)	(0.0092496)	(0.0106577)	(0.0107359)
Montana	−1.403***	−1.408***	0.246***	0.245***
	(0.0147386)	(0.0147411)	(0.0036225)	(0.003606)
Nebraska	−0.914***	−0.918***	0.401***	0.400***
	(0.0122715)	(0.0122736)	(0.0049222)	(0.0049033)
Nevada	−0.785***	−0.789***	0.456***	0.454***
	(0.0117385)	(0.0117407)	(0.0053544)	(0.0053338)
New Hampshire	−0.899***	−0.904***	0.407***	0.405***
	(0.0122086)	(0.0122111)	(0.0049671)	(0.0049463)
New Jersey	0.730***	0.738***	2.075***	2.093***
	(0.0083781)	(0.0083922)	(0.0173843)	(0.017563)
New Mexico	−1.133***	−1.137***	0.322***	0.321***
	(0.0132803)	(0.0132831)	(0.0042787)	(0.0042591)
New York	1.569***	1.628***	4.803***	5.096***
	(0.0165267)	(0.0168691)	(0.0793718)	(0.0859686)
North Carolina	0.906***	0.920***	2.473***	2.509***
	(0.0086667)	(0.0087046)	(0.0214364)	(0.0218384)
North Dakota	−1.939***	−1.944***	0.144***	0.143***
	(0.0184126)	(0.0184147)	(0.0026491)	(0.0026363)
Ohio	1.154***	1.193***	3.172***	3.296***
	(0.0123322)	(0.0125257)	(0.039113)	(0.0412842)
Oklahoma	−0.429***	−0.431***	0.651***	0.650***
	(0.0104885)	(0.0104889)	(0.0068265)	(0.0068155)
Oregon	0.0619***	0.0615***	1.064***	1.063***
	(0.009188)	(0.009188)	(0.009775)	(0.0097708)
Pennsylvania	1.299***	1.344***	3.665***	3.834***
	(0.0135686)	(0.01381)	(0.0497328)	(0.0529504)
Rhode Island	−1.374***	−1.379***	0.253***	0.252***
	(0.0145696)	(0.0145722)	(0.0036865)	(0.0036694)
South Carolina	0.0954***	0.0948***	1.100***	1.099***
	(0.0091126)	(0.0091126)	(0.0100252)	(0.0100187)
South Dakota	−1.688***	−1.698***	0.185***	0.183***
	(0.0169924)	(0.0169959)	(0.0031409)	(0.0031104)
Tennessee	0.288***	0.294***	1.334***	1.342***
	(0.0089531)	(0.0089607)	(0.0119394)	(0.0120276)
Texas	1.473***	1.534***	4.361***	4.638***
	(0.0170791)	(0.0174332)	(0.0744756)	(0.0808475)
Utah	−0.294***	−0.296***	0.746***	0.744***
	(0.0100755)	(0.0100765)	(0.0075117)	(0.0074934)
Vermont	−1.483***	−1.488***	0.227***	0.226***
	(0.0152134)	(0.015216)	(0.003452)	(0.0034355)
Virginia	0.519***	0.528***	1.681***	1.695***
	(0.0086737)	(0.0086876)	(0.0145779)	(0.0147258)
Washington	0.589***	0.596***	1.802***	1.815***
	(0.0084975)	(0.0085078)	(0.0153094)	(0.0154415)
West Virginia	−0.830***	−0.833***	0.436***	0.435***
	(0.0119205)	(0.0119224)	(0.0051993)	(0.0051809)
Wisconsin	0.254***	0.260***	1.290***	1.297***
	(0.0089887)	(0.0089953)	(0.011593)	(0.0116714)
Wyoming	−2.061***	−2.066***	0.127***	0.127***
	(0.0194199)	(0.019422)	(0.0024725)	(0.0024604)
Years fixed effects				
2000	–	0.0830***	–	1.087***
	–	(0.0070171)	–	(0.0076248)
	–			
2001	–	0.152***	–	1.164***
	–	(0.0069004)	–	(0.0080312)
2002	–	0.185***	–	1.204***
	–	(0.0068471)	–	(0.0082424)
2003	–	0.191***	–	1.210***
	–	(0.0068267)	–	(0.0082603)
2004	–	0.263***	–	1.301***
	–	(0.0067189)	–	(0.008739)
2005	–	0.347***	–	1.415***
	–	(0.0065987)	–	(0.009337)
2006	–	0.350***	–	1.419***
	–	(0.0065933)	–	(0.0093563)
2007	–	0.390***	–	1.477***
	–	(0.0065394)	–	(0.0096592)
2008	–	0.422***	–	1.525***
	–	(0.0064973)	–	(0.0099116)
2009	–	0.460***	–	1.584***
	–	(0.0064499)	–	(0.0102136)
2010	–	0.458***	–	1.581***
	–	(0.0064521)	–	(0.0102022)
2011	–	0.499***	–	1.648***
	–	(0.0064016)	–	(0.0105483)
2012	–	0.528***	–	1.695***
	–	(0.0063683)	–	(0.010793)
2013	–	0.576***	–	1.778***
	–	(0.0063132)	–	(0.0112277)
2014	–	0.632***	–	1.881***
	–	(0.006252)	–	(0.011757)
2015	–	0.678***	–	1.970***
	–	(0.006203)	–	(0.0122211)
2016	–	0.694***	–	2.002***
	–	(0.0061867)	–	(0.0123849)
2017	–	0.724***	–	2.063***
	–	(0.0061564)	–	(0.0127021)
2018	–	0.714***	–	2.042***
	–	(0.0061674)	–	(0.0125927)
2019	–	0.760***	–	2.139***
	–	(0.0061238)	–	(0.0130962)
2020	–	0.624***	–	1.866***
	–	(0.0062883)	–	(0.011737)
2021	–	0.732***	–	2.079***
	–	(0.006188)	–	(0.0128667)
Observations	n=2,325	n=2,325	n=2,325	n=2,325
Pseudo R-Square	0.932	0.935	0.932	0.935
LR-Chi^2^	1.246 × 10^6^	1.250 × 10^6^	1.246 × 10^6^	1.250 × 10^6^
d.f.	51	72	51	72

The table displays the outcomes of the Poisson regressions based on Equation 1a with and without linear time trend. Columns (1) and (2) give the structural coefficients and Columns (3) and (4)—the exponent of the structural coefficients. Standard errors are presented in parentheses. **p < 0.05, ***p < 0.01.

**Figure 1:**
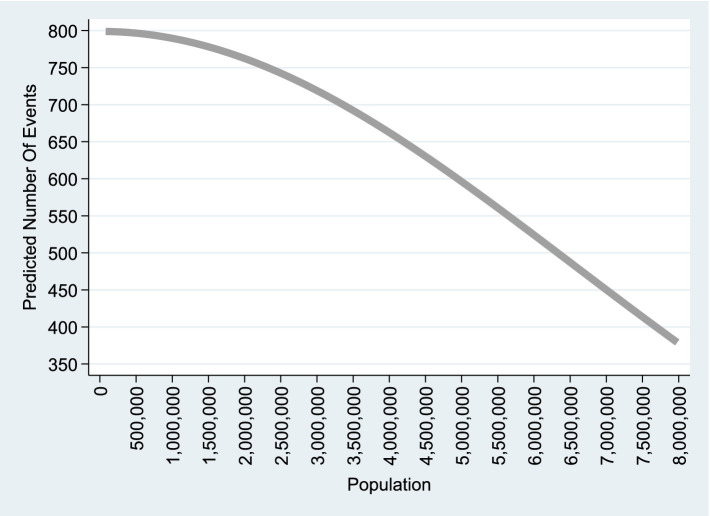
Quadratic Poisson model. This graph is based on column (1) of Table 2, which corresponds to the empirical model A1 including a linear time trend and applied to n=2,325 observations. A similar graph based on Column (2) of Table 2, representing the model without a linear time trend, is available upon request.

The results show a clear time trend in melanoma cases. Over the 23-year period, the number of new melanoma cases in the United States is projected to double $\left(1.032^{23}=2.0636\right)$ . However, as shown in Figure 1, projected new melanoma cases decrease as population size increases. For instance, there are around 800–835 projected new cases when the Caucasian population is 80,000 (the smallest state, the District of Columbia), compared to about 340–380 projected new cases when the population reaches approximately 8 million Caucasian people (the largest populated state—California). Given California’s sunny climate and high UV radiation, this outcome is particularly noteworthy. In fact, the UV index in the District of Columbia (7)—with 800–835 projected new cases per annum—is lower than that of California (10)—with only 350–370 new cases per annum.

The implication of this analysis is that the generic dummy variables for states capture variations in UV radiation levels, while population size reflects differences in medical literacy [for insights on population size and knowledge spillover effects, see, for example, O'Sullivan (16), pp. 61–62]. According to O’Sullivan: “The essential feature of knowledge spillover is that physical proximity facilitates the exchange of knowledge between people, leading to new ideas.” (p. 61).

In this context, Carlino and Hunt (17) examine, among other factors, the relationship between patent intensity and: (1) total employment, and (2) employment density (jobs per square mile). Their findings indicate that a 10% increase in total employment and employment density leads to an increase in patent intensity by 5.2% and 2.2%, respectively. Similarly, based on existing evidence, one could argue that a larger population contributes to knowledge spillover effects, which in turn foster medical literacy.

Moreover, densely populated areas are typically characterized by better health and education systems [e.g., (18)]. This, in turn, may facilitate knowledge spillover effects [e.g., (19)] and help reduce costs by utilizing the services of more skilled dermatologists and diagnosing problems at an earlier stage.

**Figure 2:**
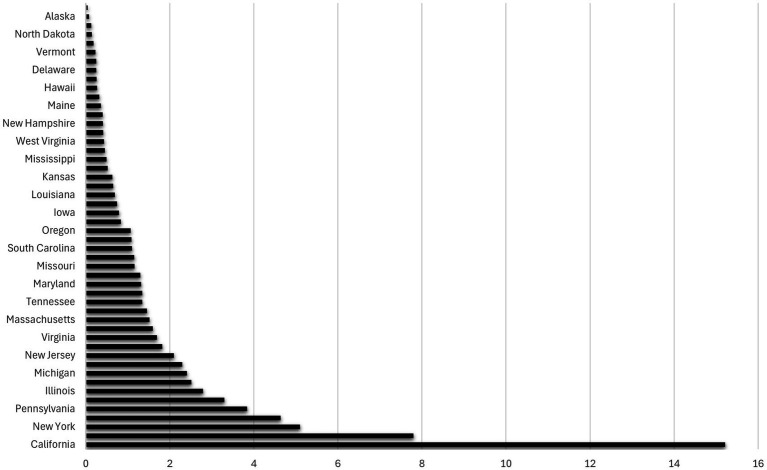
The ratio of states’ melanoma cases with respect to Alabama (the base category). The graph is based on Column (4) in Table 2. It illustrates how many times the number of melanoma cases (horizontal axis) in each state (vertical axis) surpasses the number in Alabama (the base category) where the population size of the state is controlled.

**Figure 3:**
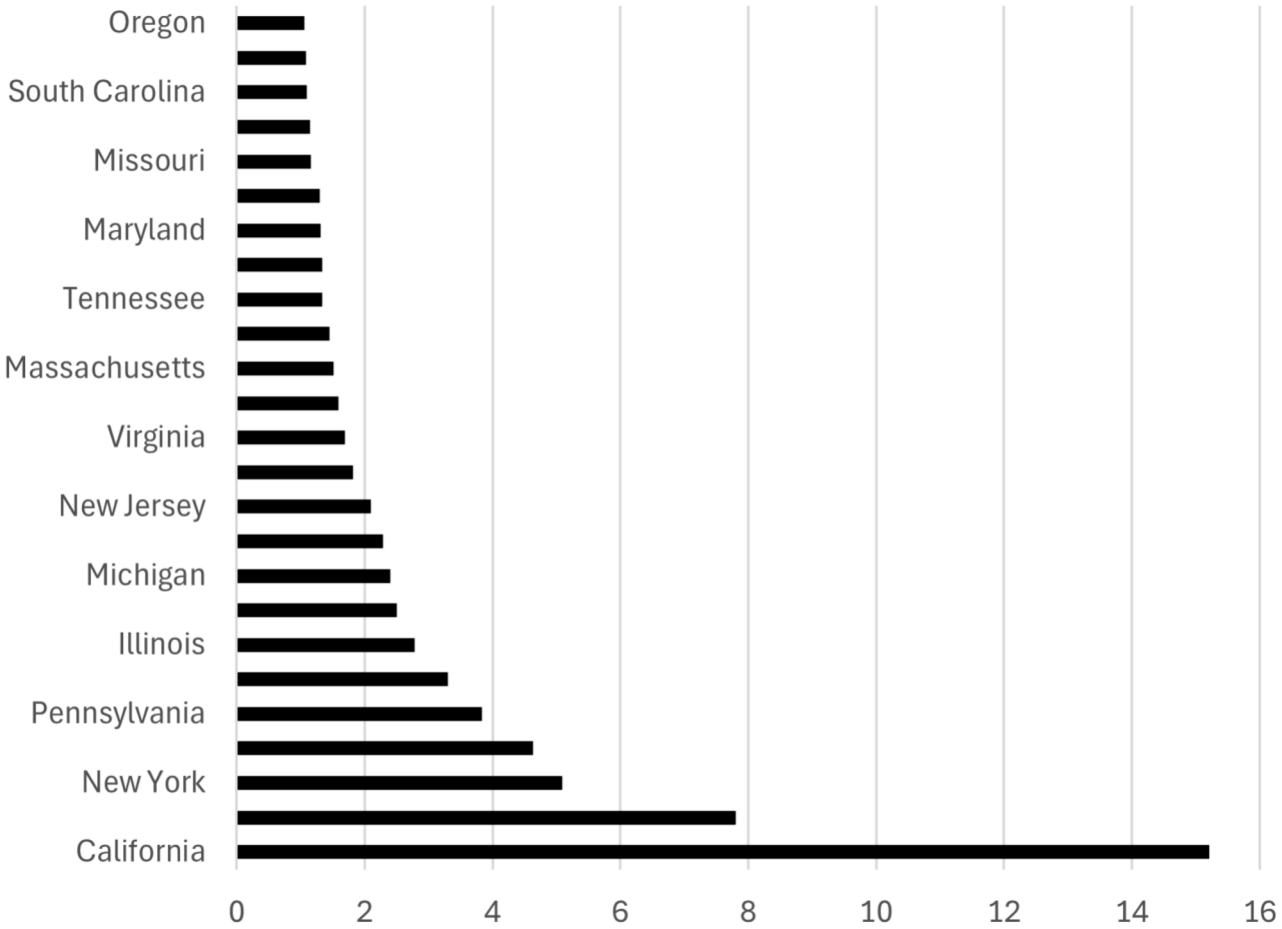
Ranking of the top 26 states by melanoma prevalence. The graph is based on Column (4) in Table 2. It illustrates how many times the number of melanoma cases (horizontal axis) in each state (vertical axis) surpasses the number in Alabama (the base category) by a factor of more than 1 where the population size is controlled.

The relationship between UV radiation and the state dummy variables is illustrated in Figures 2, 3, which are based on the Poisson regression results from Table 2. While California ranks as the state with the highest projected number of new melanoma cases when controlling for population size (13.60 to 15.22 times higher than the prevalence in the base category, Alabama), Alaska ranks lowest in terms of projected new melanoma cases when population size is controlled (0.0761 times less than the prevalence in Alabama). These figures also correspond to the UV radiation index (see Appendix A), where California ranks at the top (UV radiation index = 10) and Alaska ranks at the bottom (UV radiation index = 1).

**Figure 4:**
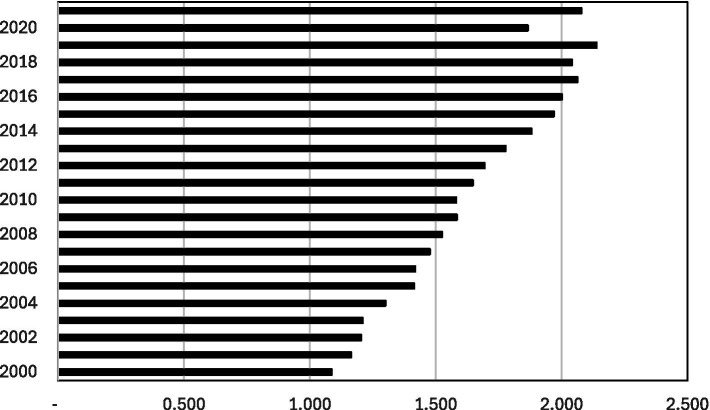
The ratio of melanoma cases of each year with respect to the base year (1999). The graph is based on Column (4) in Table 2. It illustrates how many times the number of melanoma cases (the horizontal axis) in each year (the vertical axis) surpasses the number in 1999 (the base category) by a factor of more than 1 where the population size is controlled.

Figure 4 depicts the ratio of melanoma cases for each year in relation to the base year (1999). The data for this graph is taken from column 4 in Table 2 (the non-linear time trend). It illustrates the extent to which the number of melanoma cases in each year surpasses that of 1999 (the base category) by a factor greater than 1, while accounting for population size. This graph reinforces our earlier calculation, showing that the projected number of melanoma cases has more than doubled over the 23-year period.

As a robustness check, we include Appendix B, which presents key graphical features derived from various model specifications. The table displays features from the following three graphs: (1) the graph based on Model A1 (Equation 1a), which includes only the $\text{Populatio}n^{2}$ term and is shown in Figure 1; (2) the graph based on Model A (Equation 1), the full quadratic model that includes both $\text{Populatio}n^{2}$ and Population ; and (3) the graph derived from Model A2 (Equation 1b), which includes only the Population term.

As shown in the table, all the derived graphs exhibit a decreasing trend with respect to the population variable. However, the graph in Figure 1 is concave—representing the decreasing segment of an inverted U-shaped curve—and ranges from approximately 800 projected new cases at the lowest population level to about 375 at the highest. In contrast, the graphs based on Model A (Equation 1) and Model A1 (Equation 1a) are convex—representing the decreasing segment of a U-shaped curve—and range from approximately 1,650 to 7,500 projected new cases at the lowest population level to around 0 to 375 at the highest.

## 5 Robustness tests

One concern associated with the Poisson model is the problem of overdispersion. The Cameron and Trivedi (20) overdispersion test evaluates whether variance exceeds the mean in Poisson regression. Under the Poisson assumption: $\mathrm{Var}(y_{i})=E(y_{i})=\mu_{i}$ (the population mean of the dependent variable). They propose an alternative: $\mathrm{Var}(y_{i})=\mu_{i}+\alpha \mu_{i}^{2}$ , where α is the overdispersion parameter. Rejection of the null hypothesis α=0 , supports the presence of overdispersion; non-rejection suggests the Poisson model is adequate. The test is based on an auxiliary regression using squared Pearson residuals: $r_{i}^{2}=\frac{{\left(Y_{i}-{\hat{u}}_{i}\right)}^{2}-{\hat{u}}_{i}}{{\hat{u}}_{i}}$ regressed on ${\hat{u}}_{i}$ (the residuals from estimating Model A1) excluding the constant term, If the coefficient on ${\hat{u}}_{i}$ is statistically different from zero, this indicates overdispersion [e.g., (21)].

As indicated by the descriptive statistics for the Count Case variable in Table 1, the mean (631) is close to the variance (702). However, the formal Cameron–Trivedi test for overdispersion (Table 3) rejects the null hypothesis—that the mean of the random disturbance term is zero—at the 1% significance level [e.g., (20, 21)]. This result suggests that the Poisson model may be inappropriate for addressing the research question.

**Table 3:** Overdispersion test.

Model A1 with linear time variable						
H0: equal dispersion					n =	2,325
Case Count	Coef.	Std. Err.	*t*	*p*	[95% Conf.	Interval]
$\hat{\alpha }$ (coefficient of ${\hat{u}}_{i}$ )	0.0552094	0.0011299	48.86	<0.01	0.0529937	0.0574252
					**[99% Conf.**	**Interval]**
					0.0522965	0.0581223

Model A1 with dummies for year						
H0: equal dispersion					n =	2,325
Case Count	Coef.	Std. Err.	*t*	*p*	[95% Conf.	Interval]
$\hat{\alpha }$ (coefficient of ${\hat{u}}_{i}$ )	0.0517818	0.0010887	47.56	<0.01	0.0496468	0.0539167
					**[99% Conf.**	**Interval]**
					0.0489751	0.0545885

The Cameron and Trivedi (20) overdispersion test evaluates whether variance exceeds the mean in Poisson regression. Under the Poisson assumption: $\mathrm{Var}(y_{i})=E(y_{i})=\mu_{i}$ (the population mean of the dependent variable). They propose an alternative: $\mathrm{Var}(y_{i})=\mu_{i}+\alpha \mu_{i}^{2}$ , where α is the overdispersion parameter. Rejection of the null hypothesis α=0 , supports the presence of overdispersion; non-rejection suggests the Poisson model is adequate. The test is based on an auxiliary regression using squared Pearson residuals: $r_{i}^{2}=\frac{{\left(Y_{i}-{\hat{u}}_{i}\right)}^{2}-{\hat{u}}_{i}}{{\hat{u}}_{i}}$ regressed on ${\hat{u}}_{i}$ (the residuals from estimating Model A1) excluding the constant term, If the coefficient on ${\hat{u}}_{i}$ is statistically different from zero, this indicates overdispersion [e.g., (21)].

Additional concerns include the use of total state population as an indirect proxy for medical or health literacy, which may not effectively represent it. Furthermore, state dummy variables may not sufficiently capture differences in UV radiation across states. Lastly, age is a known factor that plays an important role in melanoma prevalence.

To address these concerns, this section introduces an extended empirical model that replaces state dummy variables with a direct UV index measure from World Population. Additional control variables include a time indicator and the proportion of the population with a bachelor’s degree or higher, serving as a measure of educational attainment. The goal is to demonstrate that population size functions as a dependable proxy for medical literacy. Finally, to account for the influence of age on melanoma prevalence, the model replaces the count of melanoma cases with the annual incidence of age-adjusted melanoma prevalence.

**Figure 5:**
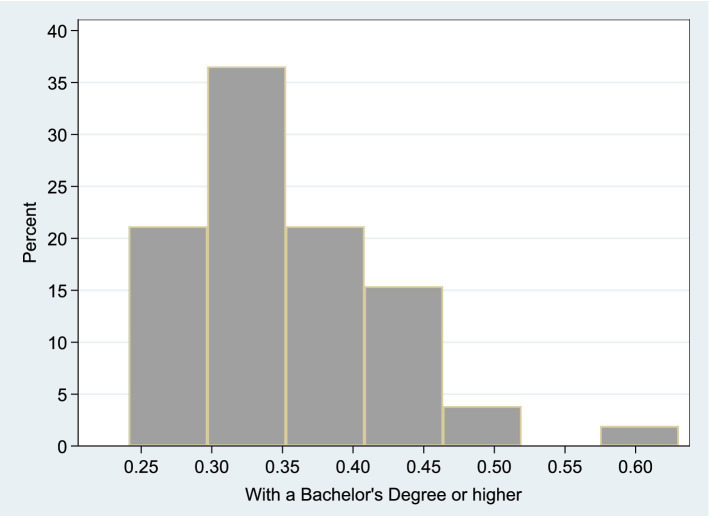
Share of educated population within each U.S. state’s total population. This histogram shows the distribution of the Pop_BA -to- Population ratio, where Pop_BA denotes the number of individuals with a bachelor’s degree or higher, and Population refers to the total state population.

Figure 5 shows the share of educated population within each U.S. State’s Total Population. This histogram shows the distribution of the Pop_BA -to- Population ratio, where Pop_BA denotes the number of individuals with a bachelor’s degree or higher, and Population refers to the total state population. According to the descriptive statistics of this variable, the median proportion of educated population is 33.81% and the mean is 34.86%. The minimum share of educated population is 24.12% (West Virginia) and the maximum is 63.05% (District of Columbia).

### 5.1 Empirical models and the two-stage least squares (TSLS) methodology

**Figure 6:**
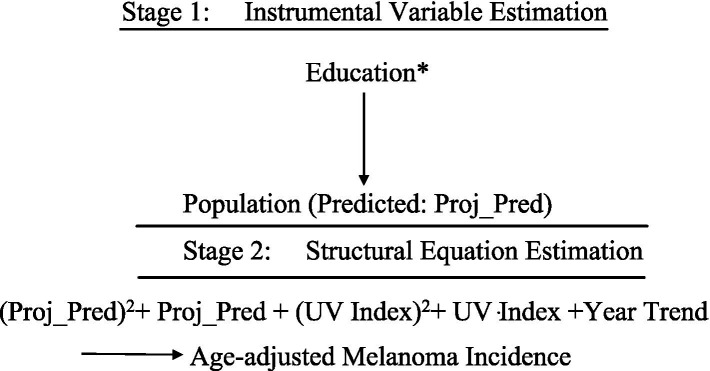
TSLS estimation of Model B1.

**Figure 7:**
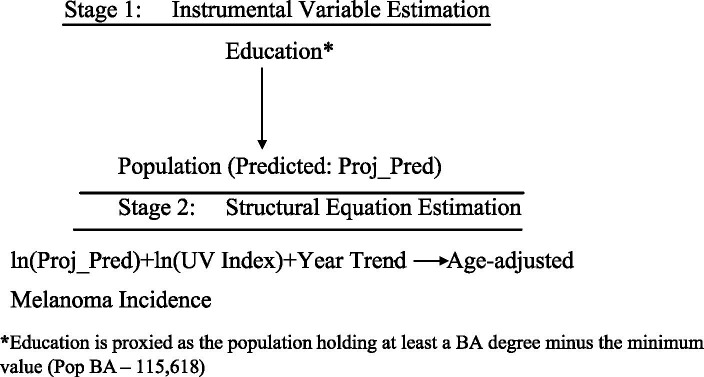
TSLS estimation of Model B2. *Education is proxied as the population holding at least a BA degree minus the minimum value (Pop BA—115,618).

Figures 6, 7 illustrate the extended models estimated using Two-Stage Least Squares (TSLS). Each model includes a main structural equation and an auxiliary first-stage regression.

*Model B*: Extended TSLS Approach

- *Model B1* Extended TSLS Approach (quadratic transformations)

(2) $$\begin{array}{c} \text{Adjusted Percent}=\beta_{1}^{\prime }\text{Populatio}n^{2}+\beta_{2}^{\prime }\text{Population}\\ +\beta_{3}^{\prime }\mathrm{UV}2020Jm^{2}+\beta_{4}^{\prime }\mathrm{UV}2020\mathrm{Jm}\\ +\beta_{5}^{\prime }+\beta_{6}^{\prime }(\text{Year}-1999)+\mu_{B1} \end{array}$$

(3) $$\text{Population}=\gamma_{1}+\gamma_{2}(\mathrm{Pop}\;\mathrm{BA}-115,618)+\in$$

- *Model B2*: Extended TSLS Approach (logarithmic transformations)

(4) $$\begin{array}{c} \text{Adjusted Percent}=\beta_{2}^{\prime \prime }\ln(\text{Population})+\beta_{4}^{\prime \prime }\ln(\mathrm{UV}2020\mathrm{Jm})\\ +\beta_{5}^{\prime \prime }+\beta_{6}^{\prime \prime }(\text{Year}-1999)+\mu_{B2} \end{array}$$

(5) $$\text{Population}=\gamma_{1}+\gamma_{2}(\mathrm{Pop}\;\mathrm{BA}-115,618)+\in$$

In both, the dependent variable is Adjusted Percent—new melanoma cases per 100,000 people, age-adjusted. Key regressors include Population, UV radiation in June 2020 (J/m^2^) (UV2020Jm), and Year. For robustness, alternate models replace Adjusted Percent with Percent, a non-age-adjusted metric.

*Model B2* also tests the joint effect of $\beta_{2}^{\prime \prime }+\beta_{4}^{\prime \prime }$ , using logs to interpret coefficients as elasticities—i.e., the percent change in melanoma incidence resulting from 1% increases in population and UV exposure.

The first-stage equation for both models instruments Population using educational attainment:

$$\text{Population}=\gamma_{1}+\gamma_{2}(\mathrm{Pop}\;\mathrm{BA}-115,618)+\in$$

With fitted values of:

Proj_Pred=476,688.2+2.7597(PopBA−115,618)

Where N=52 and $R^{2}=0.9839$

This result implies that the minimum proportion of the educated population is approximately $\frac{115,618}{476,688.2}=24.25\%$ , which aligns with the observed minimum of 24.2% in the updated descriptive statistics and histogram. Furthermore, the coefficient on the (PopBA−115,618) term indicates that for each additional educated individual, the total population increases by approximately 2.76 persons.

To address omitted variable bias from unobserved medical literacy, TSLS replaces the endogenous Population variable with Proj_Pred , ensuring consistent estimation [e.g., (9), pp. 506–536]. The second-stage equation becomes:

(2′) $$\begin{array}{c} \text{Adjusted Percent}=\beta_{1}^{\prime }\text{Proj}\_\mathrm{Pre}d^{2}+\beta_{2}^{\prime }\text{Proj}\_\text{Pred}\\ +\beta_{3}^{\prime }\mathrm{UV}2020Jm^{2}+\beta_{4}^{\prime }\mathrm{UV}2020\mathrm{Jm}\\ +\beta_{5}^{\prime }+\beta_{6}^{\prime }(\text{Year}-1999)+\mu_{B1}^{\prime } \end{array}$$

and

(4′) $$\begin{array}{c} \text{Adjusted Percent}=\beta_{2}^{\prime \prime \prime }\ln(\text{Proj}\_\text{Pred})+\beta_{4}^{\prime \prime }\ln(\mathrm{UV}2020\mathrm{Jm})\\ +\beta_{5}^{\prime \prime \prime }+\beta_{6}^{\prime \prime \prime }(\text{Year}-1999)+\mu_{B2}^{\prime } \end{array}$$

This approach clarifies the causal effect of population size and UV exposure on melanoma incidence, as schematically represented in Figures 6, 7.

### 5.2 Results

Table 4 presents the estimation results for Model B1. The variable UV2020Jm was excluded due to high collinearity with its squared term ${\left(\mathrm{UV}2020\mathrm{Jm}\right)}^{2}$ [Average Variance Inflation Factor (VIF) = 38.10]. The exclusion of UV2020Jm reduced the VIF to 4.05.

**Table 4:** Model B1.

	(1)	(2)	(3)
	Second Stage	Second Stage	First Stage
	Model B1	Model B1	Model B1
Variables	Adjusted Percent	Percent	Pred_Pop
$\text{Pred}\_\mathrm{Po}p^{2}$	1.56 × 10^−14^***	2.10 × 10^−14^***	–
	(4.82 × 10^−15^)	(6.40 × 10^−15^)	–
Pred_Pop	−4.00 × 10^−7^***	−4.44 × 10^−7^***	–
	(1.11 × 10^−7^)	(1.47 × 10^−7^)	–
$\mathrm{UV}2020Jm^{2}$	6.52 × 10^−7^***	9.81 × 10^−7^***	–
	(4.66 × 10^−8^)	(6.20 × 10^−8^)	–
Constant	13.29***	8.707***	476,688***
	(0.806)	(1.071)	(114,498.1)
(Year−1999)	0.533***	1.003***	–
	(0.0315)	(0.0418)	–
Pop_BA_min=	–	–	2.760***
(Pop_BA−115,618)	–	–	(0.0499)
Observations	n=2,284	n=2,284	n=52
R-squared	0.182	0.276	0.984
*F*-value	127.1	216.8	3,056
d.f. Numerator	4	4	1
d.f. denominator	2,279	2,279	50
Average VIF	4.11	4.11	–

The variable UV2020Jm (= UV radiation in June 2020, joules/m^2^) was excluded from Model B1 due to high collinearity with $\mathrm{UV}2020Jm^{2}$ (Average VIF = 38.10). Standard errors are presented in parentheses. **p < 0.05, ***p < 0.01.

The VIF measures how much the variance of a regression coefficient is inflated due to multicollinearity. It is calculated as:

$${\mathrm{VIF}}_{j}=\frac{1}{1-R_{j}^{2}}$$

where $R_{j}^{2}$ _is from regressing variable $X_{j}$ on all other regressors. A VIF above 5 (or 10) typically indicates problematic multicollinearity.

**Figure 8:**
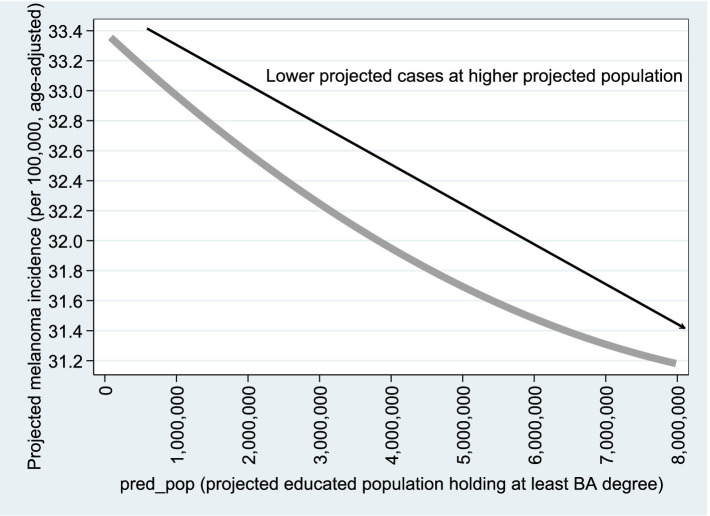
Projected Adjusted Percent vs. Pred_Pop .

**Figure 9:**
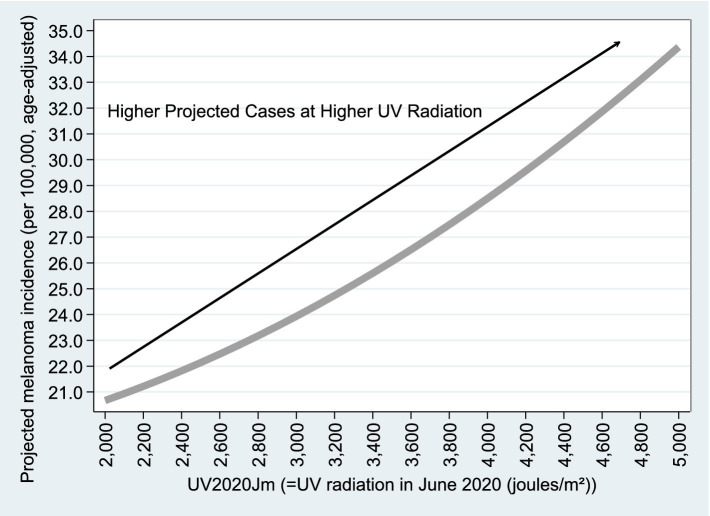
Projected Adjusted Percent vs. UV2020Jm . Figures 8, 9 are based on the estimation results from Model B1, using *n* = 2,284 observations, as reported in Column (1) of Table 4. Similar graphs based on Column (2) of Table 4 are available upon request.

Figures 8, 9 are based on Model B1 estimates using n=2,284 observations (Column 1 of Table 4); similar graphs based on Column 2 are available upon request. As shown in the figures, the relationship with population size remains consistent with the Poisson model results. For the Proj_Pred variable (projected educated population), adjusted melanoma cases drop from 28.2 to 26.0 per 100,000 as educated population increases from 0 to 100%. Regarding UV radiation, as expected, age-adjusted melanoma incidence rises from 21 to 35 cases per 100,000 as UV exposure increases from 2,000 to 5,000 $\text{joules}/m^{2}.$

Finally, Table 5 presents the results from Model B2. The low VIF value (1.05) indicates an absence of multicollinearity. According to the statistical tests shown at the bottom of the table, offsetting the projected increase in melanoma cases over the 23-year period from 1999 to 2021 requires a 12–16% rise in the educated population. Additionally, a 25–30% increase in the educated population is needed to offset a 1% increase in UV radiation.

**Table 5:** Model B2.

		(1)	(2)
		Model B3	Model B3
Variables		Adjusted Percent	Percent
ln(pred_pop)	$\beta_{2}^{\prime \prime \prime }$	−0.703***	−0.690**
		(0.224)	(0.300)
ln(UV2020Jm)	$\beta_{4}^{\prime \prime \prime }$	16.67***	24.64***
		(1.229)	(1.641)
Constant	$\beta_{5}^{\prime \prime \prime }$	−104.9***	−170.4***
		(9.716)	(12.97)
(Year−1999)	$\beta_{6}^{\prime \prime \prime }$	0.533***	1.003***
		(0.0317)	(0.0423)
Observations		n=2,284	n=2,284
R-squared		0.171	0.258
F-value		156.3	264.4
d.f. Numerator		3	3
d.f. denominator		2,280	2,280
Average VIF		1.05	1.05
Confidence Intervals			
$\;\beta_{2}^{\prime \prime \prime }+\beta_{4}^{\prime \prime \prime }$		15.97 [13.64, 18.30]	23.95 [20.84, 27.06]
$15\beta_{2}^{\prime \prime \prime }+\beta_{4}^{\prime \prime \prime }$		6.12 [−0.26, 12.50]	14.30 [5.78, 22.82]
$20\beta_{2}^{\prime \prime \prime }+\beta_{4}^{\prime \prime \prime }$		2.60 [−5.86, 11.07]	10.85 [−0.46, 22.16]
$25\beta_{2}^{\prime \prime \prime }+\beta_{4}^{\prime \prime \prime }$		−0.91 [−11.54, 9.69]	7.40 [−6.75, 21.56]
$30\beta_{2}^{\prime \prime \prime }+\beta_{4}^{\prime \prime \prime }$		−4.43 [−17.19, 8.33]	3.96 [−13.08, 20.99]
$\;\beta_{2}^{\prime \prime \prime }+23\beta_{5}^{\prime \prime \prime }$		11.55 [10.05, 13.05]	22.37 [20.37, 24.37]
$\;10\beta_{2}^{\prime \prime \prime }+23\beta_{5}^{\prime \prime \prime }$		5.22 [0.57, 9.86]	16.17 [9.97, 22.36]
$\;12\beta_{2}^{\prime \prime \prime }+23\beta_{5}^{\prime \prime \prime }$		3.81 [−1.67, 9.29]	14.79 [7.47, 22.11]
$\;14\beta_{2}^{\prime \prime \prime }+23\beta_{5}^{\prime \prime \prime }$		2.40 [−3.93, 8.74]	13.41 [4.95, 21.87]
$\;16\beta_{2}^{\prime \prime \prime }+23\beta_{5}^{\prime \prime \prime }$		0.99 [−6.20, 8.19]	12.03 [2.42, 21.64]

The estimates are based on unweighted regressions from Model B2, where the TSLS (Two-Stage Least Squares) methodology is applied. The specification is as follows: $\text{Adjusted Percent}=\beta_{2}^{\prime \prime \prime }\ln(\text{pred}\_\mathrm{pop})+\beta_{4}^{\prime \prime \prime }\ln(\mathrm{UV}2020\mathrm{Jm})+\beta_{5}^{\prime \prime \prime }+\beta_{6}^{\prime \prime \prime }(\text{Year}-1999)+\mu_{B3}$ with pred_pop (= projected educated population holding at least a BA degree) replacing the original population variable. The dependent variable, *Adjusted Percent*, represents the number of new melanoma cases per 100,000 individuals, age adjusted. *Percent* is a simplified measure calculated as $100,000\cdot \frac{\text{Case Count}}{\text{Population}}$ . 95% confidence intervals are given in square brackets. Standard errors are presented in parentheses. **p < 0.05, ***p < 0.01.

## 6 Discussion: supporting evidence for the education–melanoma link

The previous section presents robust empirical evidence supporting the claim that larger populations are associated with higher levels of medical knowledge. This is done via the following:

1. *Extension of the Empirical Model*—We augment the model to include the percentage of the educated population (defined as individuals with at least a bachelor’s degree) in each state and apply the Two-Stage Least Squares (TSLS) methodology to address potential endogeneity concerns.
2. *Incorporation of Supporting Academic Literature on Education and Medical Literacy*—Numerous studies have established a strong positive correlation between education level and health-related knowledge. For example, Cutler and Lleras-Muney (22) provide compelling evidence that higher educational attainment significantly improves health outcomes through enhanced health behaviors and literacy.
  Cutler and Lleras-Muney (22) report that, in 1990, a 25-year-old male with a college degree could expect to live an additional 54 years, while a high school dropout of the same age had a life expectancy 8 years shorter. This marked disparity in life expectancy by educational attainment is evident across all demographic groups and has remained persistent—and may even have widened—over time.
3. *Incorporation of Literature on Knowledge Spillover Effects*—Theoretical and empirical research supports the idea that knowledge can diffuse within populations, enhancing collective understanding. Glaeser et al. (23) discuss urban knowledge spillovers, while Moretti (24) demonstrates that increases in the local share of educated individuals can lead to broader productivity and knowledge gains, even among less-educated residents.
  Feldman and Audretsch (25) highlight a strong correlation between city size and innovation, noting that in 1860, the 35 largest U.S. cities had over four times more patents per capita than the national average. Today, innovation remains concentrated in major metropolitan areas, with San Francisco (8.9), Boston (8.7), New York (4.2), and Philadelphia (3.6) patents per 100,000 people.
  Further evidence of knowledge spillovers is found in patent citation patterns. Citations are five times more likely to originate from the same metropolitan area as the original patent, suggesting localized knowledge transfer. O’Sullivan (16) notes two key features of this process: first, citations cluster in the region of origin shortly after the patent is filed but gradually diffuse geographically; second, citation frequency—or “patent fertility”—varies by institution, with research universities producing the most influential patents, followed by corporate and government entities.
4. *Incorporation of Supporting Academic Literature on the Costs Associated with higher Melanoma Prevalence*—Based on the CDC report, the annual medical costs associated with treating skin cancer total $8.9 billion. Implementing proven interventions to prevent skin cancer, particularly melanoma, can save money and enhance quality of life.
  Based on a sample of 56 respondents from a popular public beach at Galveston Island, of whom 38 suffer from sunburns, Warthan et al. (26) assess the cost of sunburn by 92,720 lost workdays per annum, where the lower bound cost for lost days and treatment is $ 10 million.
  In sum, our study indicates that
  - A 12–16% increase in education level offsets the 23-year melanoma growth rate.
  - A 25–30% increase in education level offsets a 1% rise in UV exposure.
5. *Supporting Evidence for the Education–Melanoma Link*—Individuals with higher levels of education consistently demonstrate more effective behaviors for preventing melanoma, including more frequent sunscreen use, avoidance of sun exposure, and increased dermatological awareness. Falk and Anderson (27) identified education as a strong predictor of sun-safe practices among the general European population; individuals with greater educational attainment reported higher rates of sunscreen use, a preference for products with higher SPF, and a stronger willingness to enhance their sun protection habits. Similarly, Mueller et al. (28), in a study of high-risk individuals in Switzerland, found that those with higher education were more knowledgeable about melanoma risks and more likely to use sunscreen correctly—despite occasionally reporting greater sun exposure, possibly due to lifestyle factors or increased leisure time.
  This pattern is further supported empirically by Durand et al. (29), who found that vacationers with higher levels of education were more likely to engage in sun-protective behaviors, such as seeking shade and wearing protective clothing. This relationship was partly explained by their increased knowledge and more cautious attitudes toward UV exposure. Among melanoma survivors, education continued to be an influential factor; Heckman et al. (30) reported that individuals with a university-level education demonstrated higher overall sun protection scores—reflected in behaviors such as wearing hats, using sunscreen, and avoiding direct sunlight—compared to those with lower levels of education.

## 7 Summary and conclusions

Melanoma treatment costs have risen significantly, largely due to sun-induced skin damage. This study investigates how medical literacy—approximated by population size and education—affects melanoma incidence. Prior research links medical literacy, education, health systems, and population size (16–19).

Using CDC data on non-Hispanic white males and females—the most at-risk group—a quadratic Poisson regression was employed. The model incorporates annual melanoma case counts, squared population size, state dummy variables (for UV radiation), and a time trend. Results indicate a doubling of melanoma cases over the past 23 years, closely tied to UV exposure.

To address overdispersion, the extended models replaced raw case counts with age-adjusted melanoma rates per 100,000 people and substituted state-level dummy variables with direct measures of UV index and the proportion of the population with higher education. These improvements reveal that education—serving as a proxy for human capital—plays a significant role in reducing melanoma risk. Even in regions with high UV exposure, states with larger and more educated populations exhibit lower melanoma incidence rates.

The extended models indicate that a 12–16% increase in the proportion of educated individuals can offset long-term melanoma growth, while a 25–30% increase can counteract a 1% rise in UV exposure. An increase in BA-level education by 16–30 percentage points could prevent ~22,000 to ~41,000 melanoma cases in 20 years.^1^

Literature highlights disparities in early detection, especially among rural, minority, and high-risk groups, where delayed diagnoses worsen outcomes (31, 32). Access to dermatologic care remains uneven (33), and clinical training often overlooks melanoma in darker skin, particularly acral lentiginous melanoma, a subtype more common among non-white patients (34, 35). Addressing these gaps is essential for equitable outcomes.

Public policy should prioritize sun safety education and promote regular skin exams—especially in high-UV, underserved areas. Investing in health literacy today is far more cost-effective than treating preventable melanoma in the future.

### 7.1 Public policy recommendations for melanoma prevention

With global melanoma rates rising, prevention through education and early screening is vital. Health education in schools and communities can raise awareness of UV exposure, tanning, and sunscreen use. Greater health literacy promotes preventive behavior and early screening (36).

Policies should encourage routine skin checks through self-exams and clinical assessments, especially for high-risk individuals. Incentivizing providers and launching public campaigns increases screening rates (37). Regulatory measures—such as tanning restrictions and mandatory warning labels—can reduce adolescent risk (1).

### 7.2 Strengths and limitations

#### 7.2.1 Strengths

Using 23 years of state-level data (2,325 observations), the study offers robust statistical power and wide geographic coverage. Fixed-effects Poisson regression yields interpretable and credible results.

#### 7.2.2 Limitations

Population is an indirect proxy for medical literacy. A two-stage least squares (2SLS) method refines this using education levels. Overdispersion challenges the Poisson model’s assumptions but is addressed through improved modeling.

## Data Availability

Publicly available datasets were analyzed in this study. This data can be found at: https://gis.cdc.gov/Cancer/USCS/#/Trends/.

## Footnotes

^1^These estimates are derived using the formula: 197,639,521×education increase(in percentage points)×(0.703/100,000),where ${\hat{\beta }}_{2}^{\prime \prime \prime }=-0.703$ is reported in Table 5 and the 197,639,521 figure is the non-Hispanic Caucasian population in the U.S. as of 2022
