# Supplementary material for: Melanoma prevalence: can medical literacy overcome the rise in UV radiation? United States as a case study
Source: Front Public Health. 2025 Aug 6;13:1636571. doi: 10.3389/fpubh.2025.1636571 (PMC12364928; doi:10.3389/fpubh.2025.1636571)
Supplement: Supplementary file 1 [file Table_1.docx]

**Appendix A:** UV Radiation Index and Average Measure

| **State** | **UV Index** | $\boldsymbol{UV}\boldsymbol{2020}\boldsymbol{Jm=}\boldsymbol{Joules}/\boldsymbol{Meter sq}$ |
| --- | --- | --- |
| Alaska | 1 | 1957 |
| Vermont | 6 | 2873 |
| New Hampshire | 6 | 2921 |
| Maine | 6 | 2937 |
| Massachusetts | 7 | 3002 |
| New York | 7 | 3002 |
| Minnesota | 6 | 3003 |
| Wisconsin | 7 | 3005 |
| Michigan | 7 | 3021 |
| North Dakota | 7 | 3041 |
| Rhode Island | 7 | 3084 |
| Connecticut | 7 | 3100 |
| Washington | 8 | 3117 |
| Montana | 8 | 3209 |
| Iowa | 7 | 3254 |
| Pennsylvania | 7 | 3257 |
| New Jersey | 7 | 3264 |
| South Dakota | 7 | 3284 |
| Ohio | 7 | 3384 |
| West Virginia | 7 | 3484 |
| Indiana | 7 | 3486 |
| Maryland | 7 | 3503 |
| Delaware | 7 | 3512 |
| Idaho | 8 | 3604 |
| Oregon | 8 | 3607 |
| Illinois | 7 | 3615 |
| Nebraska | 7 | 3663 |
| Wyoming | 8 | 3720 |
| Kentucky | 7 | 3733 |
| Missouri | 7 | 3742 |
| Tennessee | 8 | 3859 |
| Virginia | 7 | 3937 |
| North Carolina | 9 | 4025 |
| South Carolina | 9 | 4051 |
| Mississippi | 8 | 4068 |
| Kansas | 8 | 4077 |
| Alabama | 8 | 4092 |
| Arkansas | 8 | 4103 |
| Georgia | 9 | 4168 |
| Louisiana | 8 | 4242 |
| Oklahoma | 8 | 4248 |
| Utah | 8 | 4314 |
| Nevada | 10 | 4419 |
| Colorado | 8 | 4474 |
| Texas | 9 | 4529 |
| California | 10 | 4541 |
| Florida | 10 | 4596 |
| Hawaii |  | 4793 |
| New Mexico | 9 | 4797 |
| Arizona | 10 | 4931 |

Source: World Population Review. UV Index by State 2024 Available at: <https://worldpopulationreview.com/state-rankings/uv-index-by-state> (Last Accessed on June 19, 2025).
